# Plastic recycling and their use as raw material for the synthesis of carbonaceous materials

**DOI:** 10.1016/j.heliyon.2022.e09028

**Published:** 2022-02-28

**Authors:** Rodrigo A. Muñoz Meneses, Gerardo Cabrera-Papamija, Fiderman Machuca-Martínez, Luis A. Rodríguez, Jesús E. Diosa, Edgar Mosquera-Vargas

**Affiliations:** a Centro de Excelencia en Nuevos Materiales (CENM), Universidad del Valle, Cali, Colombia; b Faculty Gama, University of Brasilia, Gama DF, 72.444-240, Brazil; c Grupo de Investigación en Procesos Avanzados para Tratamientos Biológicos y Químicos (GAOX), Escuela de Ingeniería Química, Universidad del Valle, Cali, Colombia; d Grupo de Transiciones de Fase y Materiales Funcionales (GTFMF), Departamento de Física, Universidad del Valle, Cali, Colombia

**Keywords:** Circular economy (CE), Waste plastics, Waste recovery, Nanocarbons

## Abstract

Pollution by polymeric materials - in particular plastics - has a negative effect on the health of our planet. Approximately 4.9 billion tons of plastic are estimated to have been improperly disposed of, with the environment as their final destination. This scenario comes from a linear economic system, extraction-production-consumption and finally disposal. The alarming panorama has created the need to find technological solutions that generate new uses for discarded polymeric materials or turn them into part of the production process to produce new and novel materials, such as carbon nanotubes, graphene, or other carbonaceous materials of high added value, modifying the economy for a circular and sustainable production model. This review highlights the negative impact that the disposal of plastic materials has on the environment and the research needs that allow solving the pollution problems generated in the environment by these wastes. Also, the review highlights the current and future directions of recovery plastic waste research-based to promote innovations in the plastic production sector that could allow obtaining breakpoints in other industrial sectors with the technology-based companies.

## 1 Introduction

For 2017 and 2018, the world production of plastic was 350 and 360 million metric tons, being the most abundant anthropogenic materials besides steel and concrete [1, 2]. Since the beginning of its mass production in 1950 and until 2015, around 8.300 million tons of plastic material have been produced. The popularity of plastic for everyday use is due to its high chemical stability, low density, and hydrophobicity. Apart from these properties, plastics also have an excellent strength-to-weight ratio and longevity [3]. Despite the immense social benefits that come with the use of these types of materials, for example, medical devices, life-saving protective equipment, packaging that avoids food waste, etc., it is estimated that around 5.8 billion tons, which represent 70 % of the total amount, have become waste. Of the total waste, 84 %, which is equivalent to approximately 4.9 billion tons, has been disposed of in landfills or indiscriminately in the environment (the annual flow of plastic waste in the oceans is estimated between 4.8-12.7 million tons [2]).

According to the United Nations Environment Program (UNEP) [4], the assessment of the negative externalities produced by the mismanagement of plastic waste (intensive use of non-renewable resources, degradation of natural systems, emission greenhouse gas emissions, among others) may be in the order of 40 billion USD per year. Unless current consumption patterns and plastic waste management practices change, by 2050 there will be about 12 billion tons of plastic waste (accumulated since its production in 1950) in landfills and the environment.

**Figure 1:**
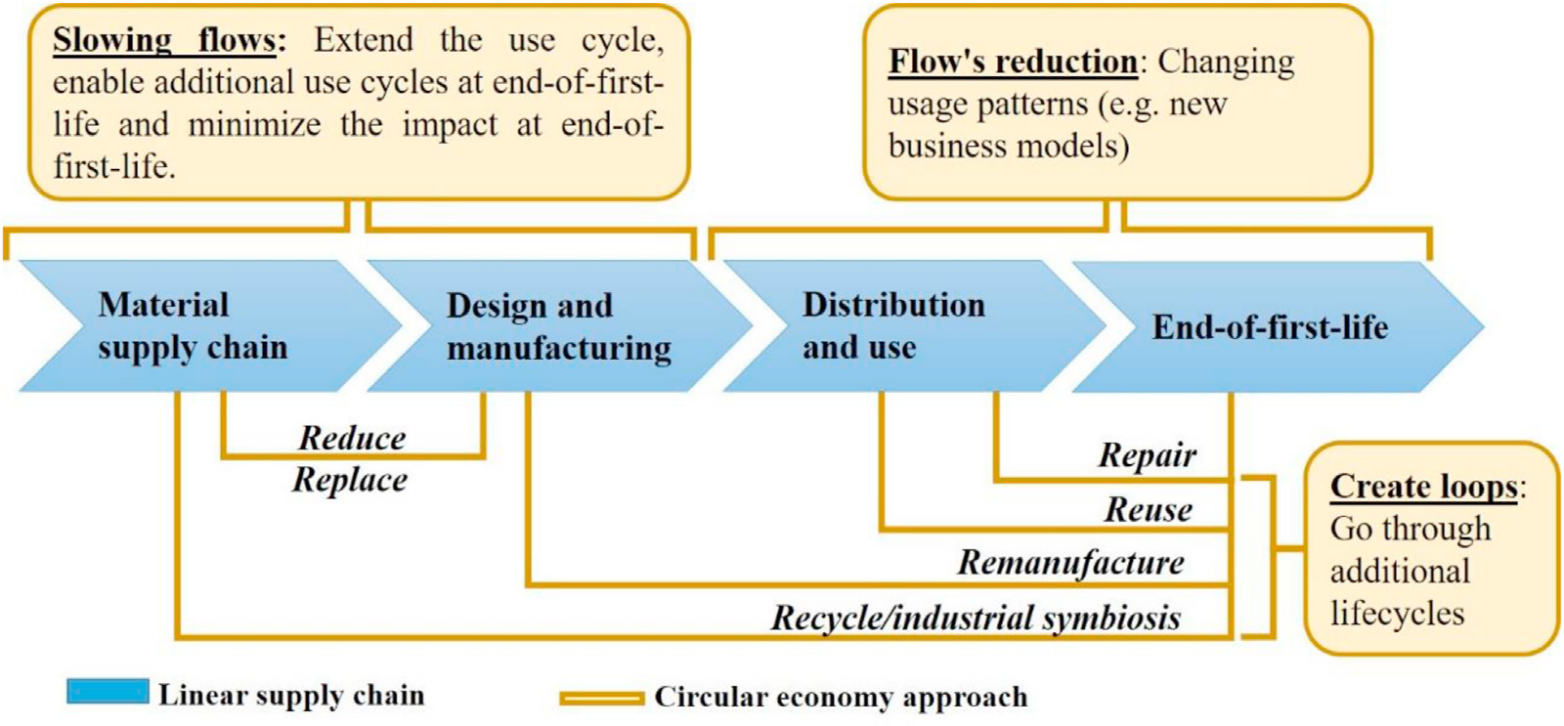
Activities of the circular economy (CE). Analysis adapted from a diagram by InnovateUK, Ref. [8].

In 2015, with the signing of the Paris Agreement on Climate Change by 195 countries [5] and the establishment of the Sustainable Development Goals (SDGs) [6], the international community recognized that, to achieve prosperous, safe and sustainable societies in the medium and long term, a transformation was necessary in the way we use natural resources and how we view “waste”, including plastic waste. This is why one of the fundamental actions to achieve compliance with these agreements comes from the change of the linear economic paradigm of extraction-production-consumption and finally disposal, which is maintained at present, towards more circular and sustainable development models, actions that are currently included in the circular economy (CE) [7]. In itself, CE is known as a systemic approach to efficiency in the use and exploitation of resources in which products and materials “at the end of their useful life” are not discarded, but rather are recycled, repaired or reused through circular value chains through three types of activities (see Figure 1), releasing added value from the resources [8].

The added value released on implementing a circular economy (CE) strategy includes social aspects (e.g., job creation, health improvement), environmental (reduction of the exploitation and use of resources as raw material, decrease in the generation of greenhouse gases, etc.), and economic (increased competitiveness of countries, decrease in waste treatment and disposal costs, etc.), as shown by various studies:

• The Waste and Resources Action Program (WRAP) in a study conducted in 2015, suggested that switching to a CE could create up to 3 million additional jobs in Europe by 2030 [9].
• A McKinsey analysis for the Ellen MacArthur Foundation (EMF) in 2015 projected that switching to a CE would bring material cost savings of up to 630 billion USD per year by 2025 in EU manufacturing sectors [10]. Similar benefits could apply to developing countries.
• A 2018 Arup study, also for the EMF, estimated that a transition to CE at scale in China could save businesses and households 10.4 billion USD by 2040, equivalent to 16 % of the projected real Gross Domestic Product (GDP) of China [11].
• Consultants such as Accenture in 2015 identified business opportunities for 4.5 billion USD by 2030 [12]. In this regard, in India alone, it is estimated that CE could create business opportunities worth 218 billion USD per year by 2030 [13].
• The Material Economics consultancy suggests that the switch to a CE could reduce CO_2_ emissions from heavy industry in the EU by up to 56 % by 2050 [14]. Along the same lines, the International Resource Panel (IRP) points out that the most efficient practices in resources could be critical to achieve the commitments of the Paris Agreement, for which it projects that the efficiency approaches of resources could reduce global greenhouse gas emissions by 60 % by 2050 [15].

According to an EMF study in 2017 [16], plastic used for the manufacture of packaging represented, in 2016, the most significant application with 26 % of the total volume of plastic produced (including PET fibers, PP, and polyamide), where the main plastic materials in this category were polyethylene (PE, 29.6 %), polypropylene (PP, 18.9 %) and polyethylene terephthalate (PET, 6.9 %). The study also reveals that most plastic packaging that circulates today is used only once, so that 95 % of its value, estimated at 80–120 billion USD annually, is lost to the economy after initial use. The same foundation in a study carried out in 2016 [17] pointed out that more than 40 years after the launch of the well-known recycling symbol, only 14 % of plastic packaging (bottles, thin films, adhesive sheets, among other formats) are collected for recycling.

The preparation of nanomaterials from plastic waste is very interesting because the environmental, economic, social, and technological aspects will have a high impact in a near future, also, the forecasting study for the nanomaterial and plastic sectors (Reports: Nanomaterials Market (2021–2026), Global Engineering Plastic Recycling Market (2021–2026), and Global Carbon Nanotubes Market (2016–2030), Mordor Intelligence) show that:

“The global engineering plastics market was estimated to be valued at 10,262.45 kilo metric ton in 2020 and is expected to reach 15,256.19 kiloton by 2026, registering a CAGR of 7.03 % during the forecast period, 2021–2026.

The major factor driving the growth of the market studied is the growing emphasis on sustainability among consumer and packaging products.

On the flipside, difficulty in collecting and sorting mixed plastic, along with the difficulty in removing residues, is expected to hinder growth of the market studied.

The global nanomaterial market (henceforth, referred to as the market studied) was reported at USD 19,928.96 million in 2020. It is expected to reach USD 57,608.26 million by 2026, at an estimated CAGR of 19.86 % over the forecast period, 2021–2026.

By structure type, the Polymeric Nanomaterials segment accounted for 43 % of the total revenue of the global nanomaterials market, in 2020. In terms of revenue, the segment was valued at USD 11,395.02 million in 2020 and is expected to reach USD 32,813.41 million by 2026 at a CAGR of 19.76 % over the forecast period.

By product type, the nanotubes segment accounted the 19.93 % of the total revenue of the global nanomaterials market, in 2020. In terms of revenue, the segment was valued at USD 13,798.02 million in 2020 and is expected to reach USD 40,134.99 million by 2026 at a CAGR of 19.95 % over the forecast period.

*In particular, the single-walled carbon nanotubes could have a value of USD 2700 per kg to 2800 per USD kg and Multi-walled carbon nanotubes USD 1000 per kg to USD 1200 per kg in 2020*”.

**Figure 2:**
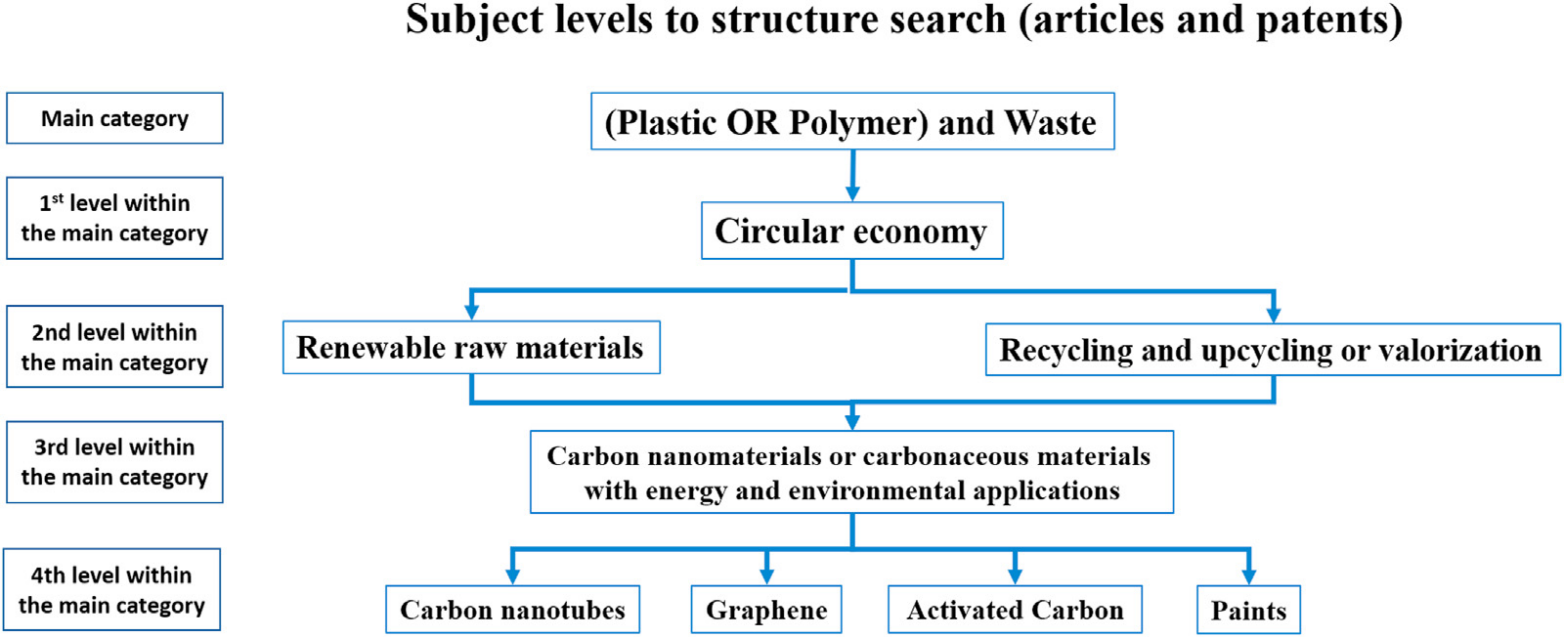
The level search strategy for scientific and technical documents.

The selected documents are related to the main topic of the review, the Scopus and WoS database were used for scientific documents and the Espacenet Orbit, and Lens database for patents. Figure 2 shows the structure used for searching the main papers and patents.

The main objective of this review is to briefly address the main impacts of plastic waste on the environment and to identify alternatives for its use by applying the principles of circular economy. This allows the identification of gaps for the generation of new knowledge and to promote innovations in the plastic production sector that could allow to obtain break points in the academic and industrial sectors for the creation of a technology-based company.

## 2 Global overview of the generation and disposal of solid waste

In the last century, the development of anthropic activities has resulted in a massive demand for energy and consumption of resources that has had a serious impact on the ecosystems and biodiversity of our planet. The high rate of increase in the world population and the per capita needs have further accentuated the problem, turning post-consumer industrial and human production waste into a global problem, both from an economic and ecological/social point of view due to costs of disposal, danger to human health, use of land for storage, contamination of land and water, climate change, etc. [18]. The World Bank indicated that, in 2016, the world's cities generated 2 billion tons of solid waste, of which at least 33 % - being extremely conservative, was not managed in an environmentally safe way [19]. With rapid population growth and urbanization, if current solid waste production and disposal habits are maintained, annual waste generation is projected to increase by 70 % from 2016 levels to 3.4 billion tons in 2050 [20].

Some figures from the most important economic blocs show the panorama described. For example, in 2016 most of the member countries of the European Union (EU) generated between 1 and 2 tons of waste (excluding the main mineral waste) per person per year [21], where the recycling rate (including composting and digestion) was only 48 % [22]. For its part, the United States in 2015 had a solid waste generation of 262.4 million tons, of which 67.8 million tons (∼ 26 %) were recycled (includes composting) [23]. In 2015, 190 million tons of municipal waste were generated in China (the world's leading producer), of which slightly more than 20 % were recycled [24]. In the above cases, more than 30 % of the solid waste ended up in landfills or garbage dumps.

Although solid waste in the widest sense (paper, cardboard, plastic, iron, etc.) is a serious problem, plastics are one of the main waste products to have received great attention in the last decade. This is because their properties allow them to remain in the environment for a long time (some take up to 500 years to decompose) and when they break down in the environment, their products (for example, microplastics) are toxic to living things when ingested. Finally, their high volume/weight ratio, as well as the great heterogeneity in composition, makes recycling not economically viable for some types of plastic [25]. The situation of plastics is detailed in greater depth below.

### 2.1 Government efforts worldwide to face the problem of solid waste

Given the huge benefits that implementing a large-scale circular economy (CE) strategy can bring, it is not surprising that the CE policy landscape has expanded markedly in the last two decades. Although CE-type thinking has existed since the 1970s, policies that explicitly refer to the circular economy with a global focus only began to be introduced in the 2000s [26]. For this reason, the countries have made different efforts and carried out cooperation at a global level for the dissemination of initiatives, principles, and exchange of practices regarding the circular economy of plastic waste in productive sectors (Tables 1 and 2).

**Table 1:** Multilateral efforts for the dissemination of the principles and exchange of practices of the circular economy in productive sectors.

Year	Productive sectors
2018	• EU CE Mission to Japan and Indonesia • EU Strategy for Plastics in a CE • New Plastics Economy Global Commitment • EU Proposal on Water Reuse • EU's Memorandum on CE Cooperation • EU CE Mission to India
2017	• World Circular Economy Forum 2017 • G20 Resource Efficiency Dialogue • First Latin American Circular Economy Forum. • EU CE Mission to Colombia • African Circular Economy Alliance • WBSCD's Factor 10
2016	• Toyama Framework on Material Cycles • EU CE Mission to Chile • EU CE Mission to China
2015	• EU Action Plan for CE • G7 Alliance on Resource Efficiency • 2030 Agenda for SDGs
2014	• EU Revised Waste Directive
2013	• Ha Noi 3R Declaration
2011	• EU Roadmap on Resource Efficiency
2008	• OECD Recommendation on Resource Productivity • Kobe 3R Action Plan

**Table 2:** Initiatives or programs launched by country within the circular economy (CE) framework.

Country	Initiative/year
United Kingdom	• UKGBC Circular Economy Programme (2018). • UK Plastics Pact (2018). • UK Industrial Strategy (2017). • UK CE Task Force (2012).
Slovenia	• Slovenia Roadmap on CE (2018).
Southern Common Market *Mercosur*	• UNIDO CE Opportunities Programme (2018)
Spain	• Spain Public Consultation on CE Strategy (2018). • Catalunya Circular (2018).
France	• France Roadmap for CE (2018).
Denmark	• Denmark CE Strategy (2018).
Scotland	• Scotland CE Investment Fund (2017).
Lao PDR	• Lao PDR CE Strategies (2017).
USA	• US Circular Economy Summit (2017). • US Sustainable Materials Management Plan (2015). • US BCSD Materials Marketplace (2015).
South Africa	• South Africa Dialogue on CE (2017).
Wales	• Wales CE Investment Fund (2017).
Turkey	• Turkey Materials Marketplace (2016).
Finland	• Finland Roadmap for CE (2016).
China	• China CE Development Strategies Plan (2013). • China Law on CE Promotion (2008). • China Law on Cleaner Production (2002).
Indonesia	• 10Y SCP Indonesia (2013).
Germany	• Germany Law on Closed Cycle Management and Waste (2012).
South Korea	• South Korea Law on Recycling Resources (2008). • South Korea Wastes Control Act (2007).
Japan	• Japan Law on Resource Efficiency (2000). • Japan Act for a Sound Material-Cycle Society (2000).

At the level of local efforts, the European Union and China have been the world leaders in this process. However, as of 2018, the national policies at the global level related to the circular economy are summarized in Table 2.

Therefore, within the multilateral efforts focused on plastic waste (Table 1), it is important to highlight the effort led by the Ellen MacArthur Foundation (EMF) in collaboration with the UN Environment Program (UNEP) in this matter through the New Plastics Economy Global Commitment program [27]. Since its launch in October 2018, the Global Commitment already unites more than 400 organizations in their common vision of a CE for plastics, keeping plastics in the economy and out of the ocean. The signatories include nearly 200 companies that are part of the plastic packaging/containers value chain, which together account for more than 20 % of all plastic packaging/containers used worldwide, including many of the major companies of the world of consumer products, retailers, and producers of plastic packaging, in addition to governments in the five continents, financial institutions, investors, and renowned university institutions.

To focus efforts at all levels, to achieve the transition of such a large and interrelated sector as plastics, from a linear economy to a circular economy scenario, the international community has outlined a vision of the sector, identifying the points that must be improved or created, as the case may be (see Ref. [25]).

Broadly speaking, the circular economy (CE) solutions in the plastic sector indicates that: a) the production of plastics from renewable sources must increase to significantly reduce dependence on fossil fuels; b) production processes and products should be redesigned to improve longevity, reuse, recyclability, as well as to avoid waste and chemical contamination; c) sustainable business models should be fostered that promote products as services, facilitate the exchange and leasing of plastic products and increase reuse; d) end-of-life plastics must be increasingly recycled into new products or into new raw materials for other industries (industrial symbiosis), in order to significantly reduce the volume of plastics that escape into the environment [25].

Other more local policies on plastic waste are those focused on single-use plastics. According to a study carried out by Tobías et al. in 2019 [28], worldwide there were approximately 160 public policies at the national and municipal level in 2018 regarding plastic bags in commerce. These policies range from bans and levies to obligations to provide information on negative environmental impact. Likewise, the prohibition of delivery of straws, stirrers, glasses, polystyrene plates, etc., in the food service industry, tourist sites, among other sectors [28, 29] are also recorded. The next section presents in more detail the advances of the applied research approaches.

### 2.2 Solutions within the framework of the circular economy for the plastics sector

Considering the problem in relation to the disposal of plastics, actions from the point of view of materials and processes in the framework of the circular economy (CE) for this plastics sector shows the current value chain of plastics of the linear type, together with the recycling and recovery actions to take it to one of circular type [30].

#### 2.2.1 Production of plastic from renewable raw materials

Renewable feedstock is primarily used to refer to feedstock of biological origin, that is, biomass, by-products derived from biomass, or carbon dioxide (CO_2_), or methane (CH_4_) derived from biological processes. It is also used to designate chemicals from CO_2_ or CH_4_ captured through man-made carbon capture and utilization processes (for example, from industrial emission gases or atmospheric carbon). Some examples of plastics in this category include but are not limited to: polyhydroxyalkanoates (PHA), polylactic acid (PLA), poly (butylene succinate) (PBS), polyethylene (PE), poly (trimethylene terephthalate) (PTT), polypropylene (PP), polyethylene terephthalate (PET), and poly (propylene carbonate) (PPC) [31]. Some plastics are in the research phase and others are already included in the formulation of commercial products (for example, PET-30 in beverage bottles) [32].

In 2017, the world production capacity of plastics from renewable raw materials was 2.1 million tons, which represented a market size of 6 billion USD [33]. However, as of 2018, the annual commercial supply of these plastic was nearly 1 % of the current total volume [34]. The proportion of non-biodegradable polymers, such as embedded bio-PE, of the total amount of bio-based plastics produced is 57 %, and the remaining 43 % is biodegradable, such as PLA and PHA. The world production capacity of these plastics is estimated to be 2.4 million tonnes in 2022 and is projected to reach a market size of 14 billion USD by 2023, at a compound annual growth rate (CAGR) of 16.5 % from 2017 [35].

The growing demand for bioplastics from the packaging industry is one of the main market drivers for these materials, representing in 2017 almost 60% of the total application market. The high growth of the packaging industry, together with the increasing regulations related to renewable packaging materials, is leading to a greater incorporation of bioplastics in relation to conventional plastics. Major players include NatureWorks (US), Braskem (Brazil), Novamont (Italy), BASF (Germany), Total Corbion PLA (Netherlands), Biome Bioplastics (UK), Bio-On (Italy), Toray Industries (Japan), Plantic Technologies (Australia) and Mitsubishi Chemical Corporation (Japan) [33].

#### 2.2.2 Recycling (post-industrial and post-consumer)

The definitions of the ASTM D5033 standard for recycling [36] include four categories: primary (mechanical reprocessing of waste materials with controlled history in products with equivalent properties), secondary (mechanical reprocessing of post-consumer materials in products requiring properties lower), tertiary (recovery of valuable chemical components such as monomers/additives or other raw materials for other industries) and quaternary (energy recovery).

##### 2.2.2.1 Primary and secondary mechanical recycling

Primary recycling, better known as re-extrusion, is used for post-industrial plastic waste management. It consists of the reintroduction in the extrusion cycle of waste, industrial plastic edges or simple polymer and pieces, to produce products similar to the original material. With technological advances, the waste generated in the transformation processes is minimal, allowing a reintroduction of more than 90 % of leftovers/surpluses in the production of plastics. This type of recycling is only feasible with semi-clean waste due to the high level of homogeneity required, so it is an option that is normally carried out within processing companies [37].

Secondary recycling refers to the reprocessing of plastic waste (mainly post-consumer) by physical means to make pellets or granules. The steps involved in this approach before manufacturing the final product are harvesting, sorting, wet cleaning, drying, shredding, pigmentation/coloring, bonding, and pelletizing/extrusion. These pellets are subsequently used in the manufacture of new, but lower quality, plastic products [38]. Lack of quality, regulations, and competitive price makes the mechanically recycled plastics difficult to compete with virgin plastics. In addition, some post-consumer plastics collected and sorted with a level of mixing and contamination that go to mechanical recycling, in combination with available processing technologies, are used primarily in new lower quality products (for example, garbage bags, containers, and basic furniture) [39]. Also, the compounds produced from recycled plastics as raw material are sold at a lower price (70–80% of the price) compared to virgin plastics [40]. This is why, to date, the economic arguments for incorporating recycled plastics in the plastic transformation chain are weak. However, in countries where the control of compliance with the regulation in the use, application of recycled material, and waste management is not so strict, it thrives as a consolidated industry with low entry barriers, which depending on its scale can be housed in low-income sectors of the population or in industrial zones. Regardless of the above, due to their impact on the quality of recycled material, technological developments to favor this type of recycling have been taking place in the stages prior to shredding (collection, classification, and cleaning) [41].

From the point of view of planning the on-site collection process and its coordination with the on-site sorting system for greater recycling efficiency, the collection bins now have sensor-based systems that can communicate in real time, indicating that type of waste they contain and how full they are [42]. Traditionally, the separation of plastics is carried out using different techniques such as separation between heavy media (density) in conjunction with hydrocyclones [43]. Other techniques include triboelectric separation [44] as well as X-ray fluorescent spectroscopy (XRF), which is suitable for flame retardant plastics [45]. However, with the increasing introduction of new polymeric materials and their combinations in different formats (e.g., multilayers), these techniques have proven to be insufficient.

In this regard, trends in the development of selection and sorting technologies include the development of automated and integrated sorting lines, comprising markers for tracking, sensor recognition, XRF compartment, online FTIR, robotics and artificial intelligence (AI). Among the many R&D projects in this area with great potential, FP7 Polymark [46] stands out, aiming to facilitate the identification of plastic waste through tracking markers for easier sorting; and FP7 SupercleanQ [47], developing quality control procedures for plastic waste. At a commercial level, in the field of robotics and AI, ZenRobotics [48] and Max-AI [49] are to the fore.

In the field of cleaning sorted plastic waste, the Spanish company Cadel Deinking [50] developed a technology to remove printing inks from plastic containers so that the recycled material is more homogeneous and closer in quality to virgin material. At the level of R&D, whether on a laboratory or pilot scale, there are other approaches for removing mechanical inks, such as particle blasting, compression vibration and cryogenic grinding; and chemical approaches such as hydrolysis through alkaline and high temperature treatment, liquid cyclone, and melt filtration [51].

Regarding processing, FP7 Ultravisc [52] stands out, in developing an ultrasonic detection technology for extrusion processes from plastic waste.

Although technological developments in relation to post-consumer classification may make the flow of plastic waste more granular/specific, if the current approach to “tailor-made” production continues with market benefits (market-pull) and not in the ease of mechanical recycling at the end of its use, in the foreseeable future it is not possible to reduce the cross contamination of materials (mixing of different grades of the same plastic and additives), which will continue to result in plastics recycled mechanically below virgin quality. Furthermore, this situation will also continue to make more than one recycling cycle not viable.

##### 2.2.2.2 Chemical recycling (tertiary)

Chemical recycling could address some limitations of mechanical recycling due to mixing, contamination, and degradation of polymers. The term chemical recycling is used to describe any advanced processing technology for converting plastic materials into smaller molecules in the liquid or gas phase using processes or chemical agents that directly affect the formation of the plastic or polymer itself. Products obtained by tertiary recycling have proven useful as fuel. However, the three main tertiary recycling types are solvent-based purification, depolymerization, and recycling of raw material, and they differ significantly in how they work and what results they produce (see Ref. [30]).

###### 2.2.2.2.1 Solvent-based purification

Generally, this process is carried out by dissolving the polymer in a specific solvent followed by removal of contaminants (pigments, additives, and other substances) by filtration or phase extraction, and then precipitating the polymer using an antisolvent in which the polymer is insoluble. The purity of the recycled polymer depends on several process parameters, and there is always the risk of residual contaminants due to the limited range of action of the solvents. However, the quality of the material is affected to a lesser extent than mechanical recycling. Because this process does not modify the polymer itself, it is susceptible to loss of properties with each reprocessing cycle. As such, this recycling method is not perpetual for plastics [53].

On a commercial scale, since 2002 the VinyLoop company processes 10.000 tons per year of PVC waste in Italy, through its patented solvent-based purification process. However, in 2018 it announced the closure of operations due to the entry into force of the European Hazardous Materials regulation [54]. In 2017, the EU-funded PolyStyreneLoop cooperative was created with the aim of recycling PS across Europe using the CreaSolv® process [55]. Unilever is currently testing this process in Indonesia, primarily to recover PE from multi-material sachets [56]. Polyolefins such as PE and PP can be purified with solvents at high temperature and pressure using commercial PureCycle Technology® [57]. In the USA, P&G has partnered with this company to purify PP residues for use in the packaging of household cleaning and hygiene products. In Europe, APK Aluminum und Kunststoffe is working on recycling various polymers (especially multilayer packaging) with its Newcycling® technology [58].

Despite the great advances in the technological development of this type of chemical recycling, a key issue is the time and energy input required for the removal of solvents, which hinders economies of scale. Furthermore, the control over the impact of the solvent on the recycled material is not clear - for example, traces of the solvent remaining in the output polymer and on the processing of the excess solvent, potentially contaminated with plastic additives and other substances [59].

###### 2.2.2.2.2 Depolymerization

Post-consumer chemical depolymerization of plastics produces the initial monomers that can be later re-polymerized into high-quality polymers or innovative small molecules that can be used as high-value-added building blocks to create unique polymeric materials or other chemicals with minimum waste. The technology generates a profitable and sustainable industrial scheme, with high product performance. This would overcome the limitation on mechanical recycling. However, because of the high stability of polymeric materials, forced conditions are generally required, such as microwave assistance [60] supercritical conditions [61] or the use of catalysts [62] to improve the efficiency of the depolymerization reactions.

Materials that have undergone research for depolymerization include the group of polyesters (PET, PLA, PHB), polyamides (PA-6 and PA-12), polyolefins (PE and PI), polyurethanes (PU), polymethyl methacrylate (PMMA), polystyrene (PS) and polycarbonate (PC) [63]. Examples of projects that have reached the pilot level on an industrial scale based on PET include GR3N [64], being part of the DEMETO consortium [65], Loop Industries [66], Ioniqa [67] and perPETual Global Technologies [68]. In the case of PA, the Aquafil company converts used nylon, that is, a PA brand, into ECONYL yarn, adding value through the sale of yarns instead of raw material [69]. Companies such as Agilyx [70] and Polystyvert [71], commercialize processes for the depolymerization of PS, although mainly for bulk applications as insulation material. In 2018, ReVital Polymers, Pyrowave, and INEOS Styrolution launched a consortium to recycle single-use PS packaging through microwave catalytic depolymerization technology [72]. In the case of PC and PMMA, their chemical recycling through depolymerization is currently limited to the research level [73].

Other advances in research on a laboratory scale are related to the development of organic catalysts to replace the traditional organometallic catalysts, since the latter are difficult to separate from the crude product, which leads to lower quality final materials. This implies high costs due to the energy intensity and the decontamination required [74]. Despite the great advances in the technological development of this type of chemical recycling, they need significant development to mature, especially if the result of depolymerization is the starting monomers and not a polymer or material with a higher added value. This scenario will remain in force at least in a near future if the prices of virgin polymers remain so affordable and competitive for the sector [75].

###### 2.2.2.2.3 Recycling of raw materials

Recycling of raw materials is distinguished by the fact that the results are simpler chemicals (for example, hydrocarbons or syngas), that cannot be converted directly into plastics, so they must be processed in several unit operations (refining-conversion-polymerization) to produce a polymer again or they can serve as raw material to manufacture other value-added products (e.g., fuels, carbon structures, among others). Within this recycling strategy, pyrolysis (thermal cracking of polymers in inert atmosphere) and gasification are distinguished. These technologies transform plastics and a vast majority of their additives and contaminants into basic chemicals. Pyrolysis is carried out by heating plastics in an atmosphere in the absence of oxygen, while gasification is carried out in an environment with a certain amount of oxygen (air in this process is used).

Pyrolysis is a generic name for all thermochemical operations that involve heating in the absence of oxygen. Since most polyolefins spontaneously degrade at only a few hundred degrees Celsius, simply adding heat is enough to break them down into smaller fragments. However, degradation is not controllable in the same way as depolymerization. Instead, bond splitting occurs at random positions, leading to a distribution of the output molecular weights and structures. It usually includes heavier waxy fragments, as well as very light fragments (C_2_–C_4_) that can be separated in the condensation stage. Such a hydrocarbon mixture resembles the composition of oil and can be used directly as fuel [76]. While pyrolysis itself is not a sufficient unit operation to chemically recycle polymers back to materials (starting monomers), the necessary additional processing infrastructure already exists in the mature and efficient value chain (chemical industry).

Almost two decades ago, pyrolysis had been tried for recycling plastic materials, but it had been discontinued several times due to economic challenges [77]. However, more recently, recognizing the limitations of each type of recycling, small and large industrial players have proposed novel and modified pyrolysis processes to increase the recycling rate of plastics [78].

Some commercial examples of this technology are the Recycling Technologies company [79], which has managed to recycle plastics that today are considered non-recyclable, such as crisp packages, multilayer films, black plastic, and mixed waste streams, turning them into fuel. Another company is APChemi, which has developed a technology for converting plastic waste into energy (fuels), including municipal plastics segregated from solid waste and multilayer packaging [80].

The benefits derived from applying this process to plastic waste are:

• It facilitates the cleaning of additives and contaminants during the process. This occurs either by converting organic additives to hydrocarbons as well or by separating the waste materials into the solid state at the end of the process.
• It is possible to some extent to control the exact composition of the hydrocarbon mixture by varying the process parameters (e.g., operating temperature, retention time, separation, reflux, etc.)
• It functions as a key complementary technology for conventional mechanical collection, sorting, and recycling.

This process on the other hand suffers from known deficiencies, such as high energy requirements, additional refinement (in case of wanting to produce polymers) and exit pollutants (PAH, dioxins, fumes, among others).

From the point of view of economic viability, the incentives for their implementation may be low, as in other chemical recycling processes, especially in countries where there is no explicit demand for the resulting materials or recycled content. However, it should be mentioned that continuous improvements are reducing energy demand, and alternative methods, such as catalytic cracking and hydrocracking (addition of hydrogen (H_2_) by chemical reaction), could increase the specificity of production and reduce the production of pollutants, while potentially requiring less energy. However, such methods require catalysts and/or a more sophisticated process setup [81].

Gasification is less sensitive to the quality of plastic waste than pyrolysis but requires more energy and large-scale operations. Gasification is a process where the materials are heated at higher temperatures, between 1000-1500 °C, in the presence of oxygen (O_2_, a limited amount) to produce synthesis gas (predominantly carbon monoxide (CO) and hydrogen (H_2_) mixture). Syngas can be used to produce a variety of plastics and chemicals (i.e., via methanol or ammonia). Both chemicals are well known versatile platforms [82]. The high temperature requirement means that gasification is energy intensive and relies on building processing units large enough to be viable. Furthermore, gasification generally needs pre-treatment to remove moisture and increase the calorific value to 14–18 MJ/kg to be sufficiently energy efficient [30].

Advances in this field are still on the laboratory scale and in some cases in the pilot stage (depending on the type of technology) with batch production. The technologies evaluated include fluidized bed reactor, fixed bed, jet beds, and plasma reactor, among others [83].

However, there are industrial-scale units for both pyrolysis and gasification, which could work more effectively by targeting certain products depending on the feedstock, market performance, and demand.

##### 2.2.2.3 Energy recovery (quaternary)

Quaternary recycling refers to the recovery of the energy content of waste through incineration. This involves burning plastic waste to produce energy in the form of heat, steam, and electricity. Currently, this is the most effective way to reduce and/or dispose of the volume of organic materials (with volume reduction up to 99 % in plastic solid waste). However, this method produces considerable toxic substances in both smoke and ash and is considered ecologically unacceptable [84]. In addition, the presence of flame retardants difficult the energy recovery process. Also, the burning processes of plastic waste produce emissions of certain polluting gases such as CO_2_, SO_x_, and NO_x_. Other environmental (i.e., heavy metals emissions) and health (i.e., carcinogenic substances) problems have been identified from the incineration or combustion processes of synthetic polymers such as PET, PS, and PE to name a few [85]. Therefore, this technology is not considered strategic in the future to help meet either the Paris Agreement or the Development Goals.

#### 2.2.3 Upcycling o valorization

Upcycling refers to the conversion of a waste material into a valuable product. It is a sustainable concept and prevents waste from being dumped directly into the landfill or incinerator, which is why it is considered a constructive approach due to the benefits of cost and waste reduction [86]. As was outlined in the previous section, the recycling that enables the closing of cycles (primary to tertiary) within the plastic sector, presents a series of advantages and disadvantages associated with its nature and the current state of development. Likewise, regardless of the time that plastic materials can be kept within the cycle resulting from the inclusion of new materials, sooner or later their reprocessing will not be technically-economically viable.

**Figure 3:**
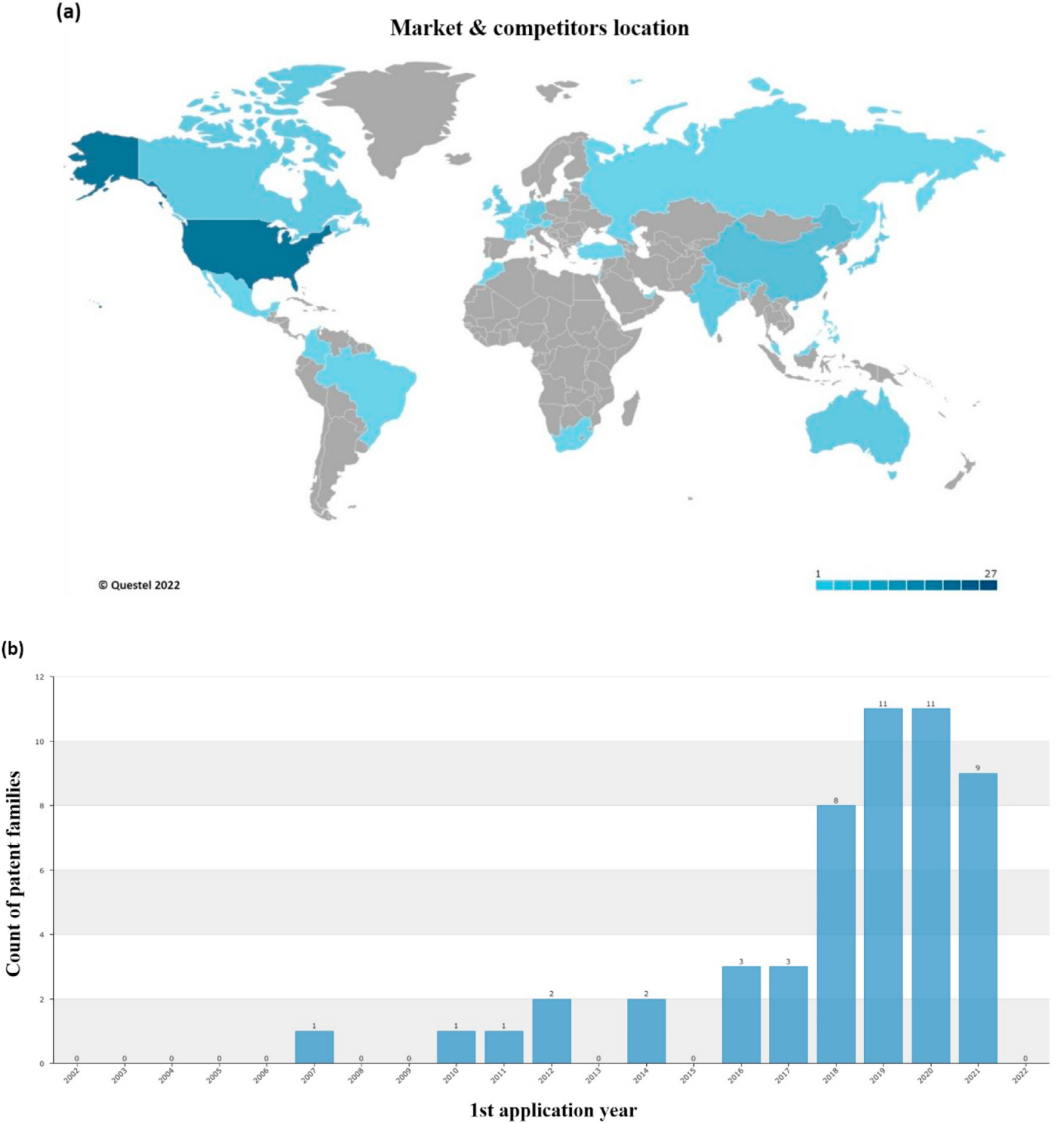
(a) Market and (b) patenting activity around upcycling of polymers and plastics.

**Figure 4:**
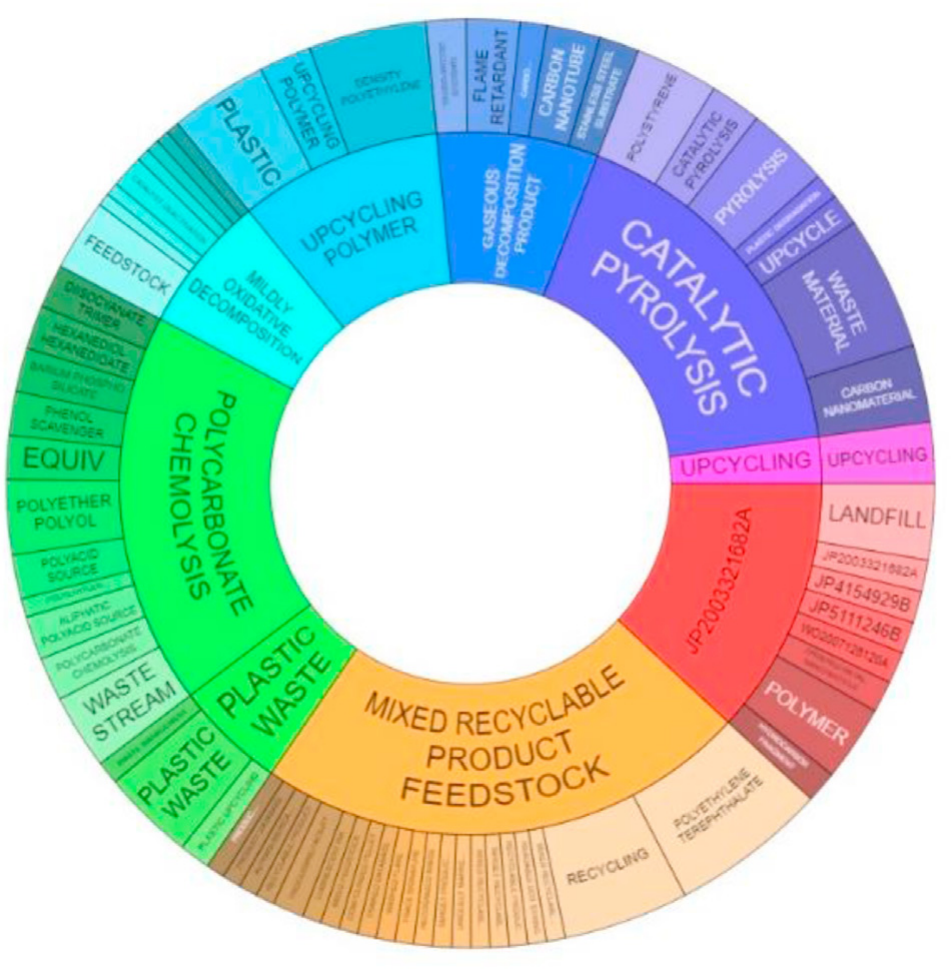
Main applications and technologies from upcycling developments.

The foregoing leads us to think that in the future a certain constant flow of plastic waste will be maintained in the environment, albeit in a lower proportion than is currently the case. Therefore, to complement the technological offer in the use of plastic waste, in recent years scientific interest has turned towards the development of recycling solutions with greater added value, mainly in synergy with other value chains (see Ref. [30]) and which is related to the number of patents and scientific articles related to the upcycling of polymers or plastics. Figure 3 presents the evolution of patents and their interest on the part of applicants worldwide. In addition, Figure 3 shows the economic and scientific interest and the possible impact of the technologies associated with the use of plastic waste. Also, Figure 4 shows the main technologies and possible applications related with the upcycling technologies. Some reported examples are [38]:

o Production of carbon nanomaterials (multiple walls, hollow spheres, threads, among others) for potential use in various applications (environment, energy, health, aerospace, etc.).
o Hollow spherical carbon from polystyrene foam spheres and pyrolysis of sludge, which are then used as adsorbents to treat wastewater.
o Conversion of expanded PS waste into polymeric azo dyes with sulfonamide group for the pigment industry.
o Preparation of doped carbon catalyst for oxygen reduction using PS foam, melamine and iron chloride as precursors.
o Modification of ordinary paving asphalt, using, for example, a carbon fiber waste packaging based on PE and polyacrylonitrile over ordinary polymer modifiers.
o Adsorbents to remove lead from wastewater using PS waste.
o High-surface composite material for CO_2_ adsorption, from amine-functionalized PVC waste.
o Hyper-cross-linked polymers for CO_2_ capture, using the Friedel-Crafts reaction with expanded PS foam as raw material and 1,2-dichloroethane as solvent and cross-linking agent.
o Substitution of constituents in concrete mixtures.

##### 2.2.3.1 Production of carbon nanomaterials from plastic waste

Of the valorization approaches available at the international and national level, the production of carbon nanomaterials presents the greatest potential. This is because: 1) by themselves carbon nanomaterials represent a growing market segment (marketed as raw material), and 2) they can be part of different products that address other markets (environment, energy, health, aerospace, others), with growth in demand.

Due to the fact that carbon is the main component of plastics, such is the case for example of polyethylene (85.6 % wt of carbon), polypropylene (85.6 % wt of carbon), polystyrene (92.2 % wt of carbon), terephthalate made of polyethylene (62.6 % wt carbon), and polyacrylonitrile (67.9 % wt carbon), plastic waste can therefore provide a carbon source to produce carbon nanomaterials [87]. Table 3 shows the research and patenting activity related to upcycling of plastic waste to carbon nanomaterials.

**Table 3:** Research and patenting activity related to upcycling of plastic waste to carbon nanomaterials in the period 2000 to 2019.

Search criteria	Activity in research	Activity in patenting
*Title, Abstract, Keywords/claims*	*Database: SCOPUS*	*Database: Lens Org.*
Reuse, valorization, upcycling, value added, waste, residual, plastic/polymer + carbon nano	31 scientific publications (As of 2010, publications on the subject begin)	10 patent documents (since 2007)

Although there are several structures, those with a defined market will be briefly highlighted.

###### 2.2.3.1.1 Carbon nanotubes (CNTs)

These are rolled graphene sheets that form a cylindrical piece and depending on how the graphene sheets are rolled, they can display different properties [88]. The attractive properties of CNTs are essentially divided into five categories: (i) electrical: semiconductor or metallic behavior, 100 times more conductive than copper; (ii) mechanical: very high tensile strength (100 times greater than steel); (iii) thermal: high thermal stability and thermal conductivity; (iv) chemical: chemically inert while being related to chemical/biological functional groups; (v) structure: ideal one-dimensional (1D) system with anisotropic properties, with extremely high aspect ratios [89].

The applications of this nanomaterial include electronic, photovoltaic, electrodes, transport, remediation, and medical devices, among others. As of 2018, the market size was 4.55 billion USD, and it is projected that by 2023 it will be 9.84 billion USD, at a CAGR of 16.70 % during the forecast period. To date, more than 100 companies are producing and marketing of CNTs [90].

Since its discovery in 1991, a great deal of research and patenting activity has been reported for this nanomaterial, as shown in Table 4.

**Table 4:** Research and patenting activity related to carbon nanotubes in the period 1991 to 2019.

Search criteria	Activity in research	Activity in Patenting
*Title, Abstract, Keywords/claims*	*Database: SCOPUS*	*Database: Lens Org.*
Carbon nanotubes	109,631 scientific publications	37,752 patent documents grouped by family

There are four main methods to produce CNTs commercially: arc discharge, laser ablation, chemical vapor deposition (CVD), and combustion (flame) synthesis. Following the discovery of multi-walled and then single-walled carbon nanotubes by electric arc carbon vaporization, several varieties of this technique were described, such as metal doping, different electrode configurations, or different types of carbon sources [86]. Given that in the traditional process in the chemical industry 60 %–90 % of production costs come from raw materials, being able to successfully incorporate inexpensive raw materials into the production of CNTs has become a challenge [91].

In this regard, several strategies have been proposed to prepare CNT from plastic waste, for example, combined catalysts, high pressure autoclave, pyrolysis-gasification-carbonization, pyrolysis-combustion-carbonization, and CVD method. Meanwhile, the materials that have been most extensively studied are polypropylene (PP), polyvinyl chloride (PVC), low-density polyethylene (LDPE), and polystyrene (PS). However, important production yields that allow it to move to a pilot stage have not yet been achieved. Moreover, most studies use costly nickel-based catalysts, hence the need to find other catalyst options [92].

###### 2.2.3.1.2 Graphene

Graphene is a single, two-dimensional sheet of carbon atoms arranged in a hexagonal lattice. It was discovered in 2004 through the adhesive tape peeling process [93]. The ideal graphene sheets are highly ordered and exhibit a high Young's modulus (1 TPa), high thermal conductivity (5000 W/mK) and high electron mobility (>200,000 cm^2^/Vs), a high specific surface area (up to 2630 cm^2^/g), optical transmittance (up to 97.7%), chemical stability and quantum Hall effect at room temperature. Additionally, graphene is 200 times stronger than steel, making it one of the strongest materials ever tested [94]. Due to its impressive characteristics, graphene is used in a wide range of applications, mainly in photonics, electronics, biomedical and environmental pollution control as a biosensor, drug carrier, energy storage, nanocomposite polymers and adsorbent [95].

The global size of the graphene market was 42.8 million USD in 2017, and from that date to 2025 a CAGR of 38.0 % [96] was projected, to reach a market size of 552.3 million USD. To date, more than 20 companies produce and market this material [97]. Since its discovery, this nanomaterial has reported great activity in research and patenting, as shown in Table 5.

**Table 5:** Activity in research and patenting related to the graphene topic in the period 2004 to 2019.

Search criteria	Activity in research	Activity in patenting
*Title, Abstract, Keywords/claims*	*Database: SCOPUS*	*Database: Lens Org.*
Graphene	121,979 scientific publications	59,359 patent documents grouped by family

At the commercial level there are four main methods to produce different types of graphene. Micromechanical exfoliation, chemical vapor deposition (CVD), reduction of the liquid phase of graphene oxide and epitaxial growth are the four different synthetic methods of graphene [95]. These methods use traditional hydrocarbons as raw material that make the process difficult to control and thus fail to achieve its scaling potential. As such, the advantage of polymers as precursors over traditional hydrocarbons in preparing graphene lies in the easy control of the total number of carbon atoms. Hence the need to develop a method that allows the successful incorporation of plastic waste as raw material [98].

Considering this, several strategies have been proposed using the CVD method to prepare different types of graphene from plastic waste, these being polyethylene terephthalate (PET), polyethylene (PE), polyvinyl chloride (PVC), polypropylene (PP), polystyrene (PS) and polymethylmethacrylate (PMMA) [99]. However, the yield of graphene is low, and the size of the graphene is limited by the size of the metal substrate. Therefore, there is a need to develop new approaches for the efficient conversion of plastic waste to graphene with high yields and controllable layers (single layer, bilayer, or a few layers) [100].

###### 2.2.3.1.3 Activated carbon

Activated carbon is a carbonaceous material that has a high surface area. Its internal structure is composed of micropores, so these can adsorb a wide variety of substances, i.e., they can attract molecules to their internal surface and therefore act as an adsorbent [101]. The properties of this material depend on the material of its origin and its structure may be different depending on the raw material from which it was made. Industrially they are produced through reaction with gases, by physical activation, or through the addition of chemicals in the process known as chemical activation. Activated carbon is made up of primary microcrystals and is composed of structures of hexagonal planes of two-dimensional carbon atoms that lack crystallographic order in the direction perpendicular to the sheets. They therefore present a high percentage of the highly disordered structure. Activated carbon is characterized by a branched pore system, in which pores of different sizes, such as mesopores (d = 2–50 nm), micropores (d = 0.8–2.0 nm), and sub-micropores (d ≤ 0.8 nm) branch from macropores (d ≥ 50 nm). The pore volume of activated carbons is generally greater than 0.2 ml/g. The internal surface area is generally greater than 400 m^2^/g. Pore width varies from 0.3 to several thousand nanometers [97].

The conversion of plastic waste into carbon materials is generally prepared by pyrolysis, in batch or continuous mode, because this process is well known, the focus is can find the optimal operations conditions for different materials with specific properties according to the applications required. Vieira et. al., [102] and Wijesekara et. al., [103] show complete reviews about the preparation methods for carbon-based materials from plastic waste. These reviews verified that the different conditions (temperature, catalysts, flow, type of atmosphere, i.e.), used to obtain the materials could be used to find different properties. Fuks et. al., [104] show a study for the synthesis of activated carbon from PET waste by pyrolysis under N_2_ environmental; also, Ilyas et. al., [105] produce activated carbon from PETs bottles under controlled conditions (inert atmosphere at high temperature). Therefore, the operational conditions could be modified according to the specific structure, then the rapid, medium, and high pyrolysis could produce different activated carbons with different properties.

Activated carbon has a wide range of applications both in its powder presentation and in its granular presentation in liquid and/or gaseous medium. In general, pulverized carbon is applied in a liquid medium while granulated activated carbon can be applied in both environments. Standing out among its applications in a liquid medium are: discoloration of sugar liqueurs, purification of water (elimination of odor, color, chemical substances, bacteria), wastewater treatments, water dichlorination for use in the manufacture of soft drinks, decolorization and enhancement of alcoholic beverages (wines, rums), purification of edible fats and oils, protein purification, as a medicine in the detoxification of people, purification of blood plasma, and separation of metallic elements (gold, silver). In a gaseous medium, its applications are also abundant, among which are: storage and separation of gases, gas masks, anti-radioactive protection in nuclear plants, deodorizing of food products. In addition, today it has broad application prospects as a catalytic support and as a catalyst [106].

The size of the global activated carbon market was estimated at 4.72 billion USD in 2018. This market is expected to expand at a CAGR of 17.5 % during the following years, mainly due to its increasing demand in water treatment applications and wastewater treatment [107]. Since its discovery, this material has reported great research and patenting activity, as shown in Table 6.

**Table 6:** Activity in research and patenting related to the activated carbon topic in the period 2004 to 2020.

Search criteria	Activity in research	Activity in patenting
*Title, Abstract, Keywords/claims*	*Database: SCOPUS*	*Database: Lens Org.*
Activated carbon	100,027 scientific publications	3,178,257 patents

To produce activated carbon, the raw material is required to have a porous structure, adequate mechanical resistance, null or low inorganic material, high carbon content and, most importantly, the raw material must be abundant. Coal, wood, lignite, coconut shells, walnuts and almonds, olive seeds and some polymers have traditionally been used as raw materials [108]. It is for this reason that there is a need to develop methods that allow the successful incorporation of plastic waste as a raw material to obtain activated carbon, considering that this waste is abundant and has a high carbon content.

###### 2.2.3.1.4 Paints

Paints are liquid compositions that turn into a solid film after being applied in thin layers to a surface. The paints dry forming an adapted film on the applied surface. They are compact and well adhered. They are applied to hide the primitive appearance of the surface, giving it color, a different look, as well as having the aim of lending protection from attack by external agents [109].

Paints generally have several basic components: the vehicle, the pigment, additives, etc. The vehicle, also known as an emulsifier, dissolves in a medium to form the liquid part of the paint (the part that is polymerized). This provides homogeneity and the protective film. In addition, it controls the sliding properties of the coating and helps improve its hardness and resistance. Pigment, meanwhile, is an organic or inorganic compound whose mission is to provide paint with color and coating power [110]. The additives cover many products that are generally added to paints for specific purposes. These are generally products added in small amounts to cause or attribute certain effects or properties that would not be achieved with only the vehicle, pigments and/or solvents [111].

Paints have many applications and their commercialization is considered an important economic activity. Their global market is expected to exceed a volume of 167 trillion dollars in 2022 mainly due to increasing urbanization and industrialization, according to the TechSci Research report entitled Global paints and coatings market by technology, by application, by region, competition forecast & opportunities, 2012–2022. Due to the growing demand for the use of these products, it is also considered a viable alternative to include plastic waste in a new production cycle, generating added value.

## 3 Conclusions

Plastic waste is highly resistant to degradation and causes a range of environmental concerns associated with the accumulation of waste in nature, since in the long term it exerts toxic effects on living beings, soils, and water sources. To date, the recycling of plastics has not been an economically viable solution and it is estimated that most household plastics end up being improperly disposed of in landfills or outdoors after their first use. As such, this review demonstrates that it is possible to incorporate plastic waste into production processes that allow the production of carbonaceous materials with high added value, constituting a sustainable alternative for the final disposal of this waste. However, it should be stressed that the methods for obtaining these materials are still in the research phase or the laboratory, so it remains a challenge to take it to an industrial scale and make it economically viable. Among the most popular carbonaceous materials, for their electrical, mechanical, thermal, chemical, and structural properties, carbon nanotubes stand out. As of 2018, the market size was 4.55 billion USD, and it is projected that by 2023 it will be 9.84 billion USD, which is why using recycled plastics as raw material is considered a good production option. Therefore, partnerships among scientists and engineering are essential for integrating such processes with existing manufacturing methods to allows for conversion of post-consumer products with high value-added.

Other products can also be suggested to use raw material from recycled plastics, in particular graphene, activated carbon and paints. All these economic activities would introduce plastic waste into a new production process, generating added value to this type of waste.

The new applications on energy, environment, and healthcare are the driving force for the scientific and technological development of nanomaterial from plastic waste. The hot pots are wastewater treatment, energy conversion, CO_2_ conversion, fuel production, energy storage, and controlled drug delivery.

## Declarations

### Author contribution statement

All authors listed have significantly contributed to the development and the writing of this article.

### Funding statement

Edgar Mosquera was supported by Minciencias (BPIN 2020000100377).

Jesús Diosa was supported by Universidad del Valle (Strengthening of Centers and Institutes).

### Data availability statement

No data was used for the research described in the article.

### Declaration of interests statement

The authors declare no conflict of interest.

### Additional information

No additional information is available for this paper.
